# Relative validity of a short screener to assess diet quality in patients with severe obesity before and after bariatric surgery

**DOI:** 10.1017/S1368980022001501

**Published:** 2022-07-04

**Authors:** Laura Heusschen, Agnes AM Berendsen, Michiel GJ Balvers, Laura N Deden, Jeanne HM de Vries, Eric J Hazebroek

**Affiliations:** 1 Vitalys Obesity Clinic, Part of Rijnstate Hospital, Arnhem, 6800 TA, The Netherlands; 2 Divison of Human Nutrition and Health, Wageningen University, Wageningen, The Netherlands

**Keywords:** Screener, Diet quality, Validity, Reproducibility, Obesity, Bariatric surgery, Weight loss surgery

## Abstract

**Objective:**

To determine the relative validity and reproducibility of the Eetscore FFQ, a short screener for assessing diet quality, in patients with (severe) obesity before and after bariatric surgery (BS).

**Design:**

The Eetscore FFQ was evaluated against 3-d food records (3d-FR) before (T0) and 6 months after BS (T6) by comparing index scores of the Dutch Healthy Diet index 2015 (DHD2015-index). Relative validity was assessed using paired *t* tests, Kendall’s tau-b correlation coefficients (*τ*b), cross-classification by tertiles, weighted kappa values (*k*_*w*_) and Bland–Altman plots. Reproducibility of the Eetscore FFQ was assessed using intraclass correlation coefficients (ICC).

**Setting:**

Regional hospital, the Netherlands.

**Participants:**

Hundred and forty participants with obesity who were scheduled for BS.

**Results:**

At T0, mean total DHD2015-index score derived from the Eetscore FFQ was 10·2 points higher than the food record-derived score (*P* < 0·001) and showed an acceptable correlation (*τ*b = 0·42, 95 % CI: 0·27, 0·55). There was a fair agreement with a correct classification of 50 % (*k*_*w*_ = 0·37, 95 % CI: 0·25, 0·49). Correlation coefficients of the individual DHD components varied from 0·01–0·54. Similar results were observed at T6 (*τ*b = 0·31, 95 % CI: 0·12, 0·48, correct classification of 43·7 %; *k*_*w*_ = 0·25, 95 % CI: 0·11, 0·40). Reproducibility of the Eetscore FFQ was good (ICC = 0·78, 95 % CI: 0·69, 0·84).

**Conclusion:**

The Eetscore FFQ showed to be acceptably correlated with the DHD2015-index derived from 3d-FR, but absolute agreement was poor. Considering the need for dietary assessment methods that reduce the burden for patients, practitioners and researchers, the Eetscore FFQ can be used for ranking according to diet quality and for monitoring changes over time.

Obesity is reaching epidemic proportions, and bariatric surgery (BS) is proven to be one of the most effective treatments, resulting in substantial and long-term weight loss and improvement of obesity-related comorbidities^(1–3)^. BS is performed in individuals with a BMI above 40 kg/m^2^ or a BMI above ≥ 35 kg/m^2^ with obesity-related comorbidities such as diabetes mellitus type 2, hypertension, obstructive sleep apnoea and dyslipidaemia^(4)^. Worldwide, the Roux-en-Y gastric bypass (RYGB) and sleeve gastrectomy are the most commonly performed bariatric procedures^(5)^.

After BS, the amount of food that can be ingested is significantly reduced, resulting in a lower energy intake^(6)^. Additionally, food intolerances after surgery may lead to avoidance of food groups which in turn may impact diet quality^(7)^. Poor diet quality is consistently reported in patients with (severe) obesity, including those presenting for BS^(8–10)^. This could impact their risk of developing nutritional deficiencies as well as the success of their weight loss after surgery^(10–12)^. Therefore, monitoring diet quality is an important component in the BS programme.

Diet quality can be assessed with the Dutch Healthy Diet index 2015 (DHD2015-index)^(13)^. The DHD2015-index measures adherence to the Dutch food-based dietary guidelines published in 2015 by the Health Council of the Netherlands^(14)^. The DHD2015-index can be calculated using data from multiple food records, 24-h dietary recalls or a single FFQ. Unfortunately, these methods are time-consuming and burdensome and therefore less likely to be used in everyday clinical practice. For this reason, a short screener, the Eetscore FFQ, was developed to estimate the DHD2015-index in time-limited situations. The Eetscore FFQ showed to be acceptably correlated with the DHD2015-index derived from a full-length FFQ in a normal-weight adult population^(15)^. However, the Eetscore FFQ has not been evaluated in patients with (severe) obesity before or after undergoing BS.

Accurate measures of diet quality are needed to optimise nutritional care provided to these patients during the BS programme, but validated dietary assessment tools in this specific population are lacking^(16)^. Therefore, this study aimed to evaluate the relative validity and reproducibility of the Eetscore FFQ as a screener for diet quality in patients with (severe) obesity before and 6 months after BS.

## Methods

### Study design and participants

Between October 2018 and September 2019, patients with obesity who were eligible and scheduled for BS at Vitalys Obesity Clinic, part of Rijnstate hospital (Arnhem, the Netherlands), were asked to participate in this prospective cohort study. Participants were included approximately 6 weeks pre-surgery (T0) and followed up until 6 months post-surgery (T6). Exclusion criteria were a non-Dutch eating pattern, suffering from an eating disorder, inability to fill in questionnaires or food records and a previous bariatric procedure other than an adjustable gastric band.

In total, 200 participants signed the informed consent and were included in the study. Both before and after BS, we evaluated the Eetscore FFQ against 3-d food records (3d-FR) as reference method by comparing index scores of the DHD2015-index derived from both methods. At both time points, demographic information was collected and participants were asked to complete the Eetscore FFQ, followed by a 3d-FR as reference method. At T0, the Eetscore FFQ was completed twice (Eetscore FFQ1, Eetscore FFQ2) with an interval of approximately 5 weeks in order to analyse reproducibility.

**Fig. 1:**
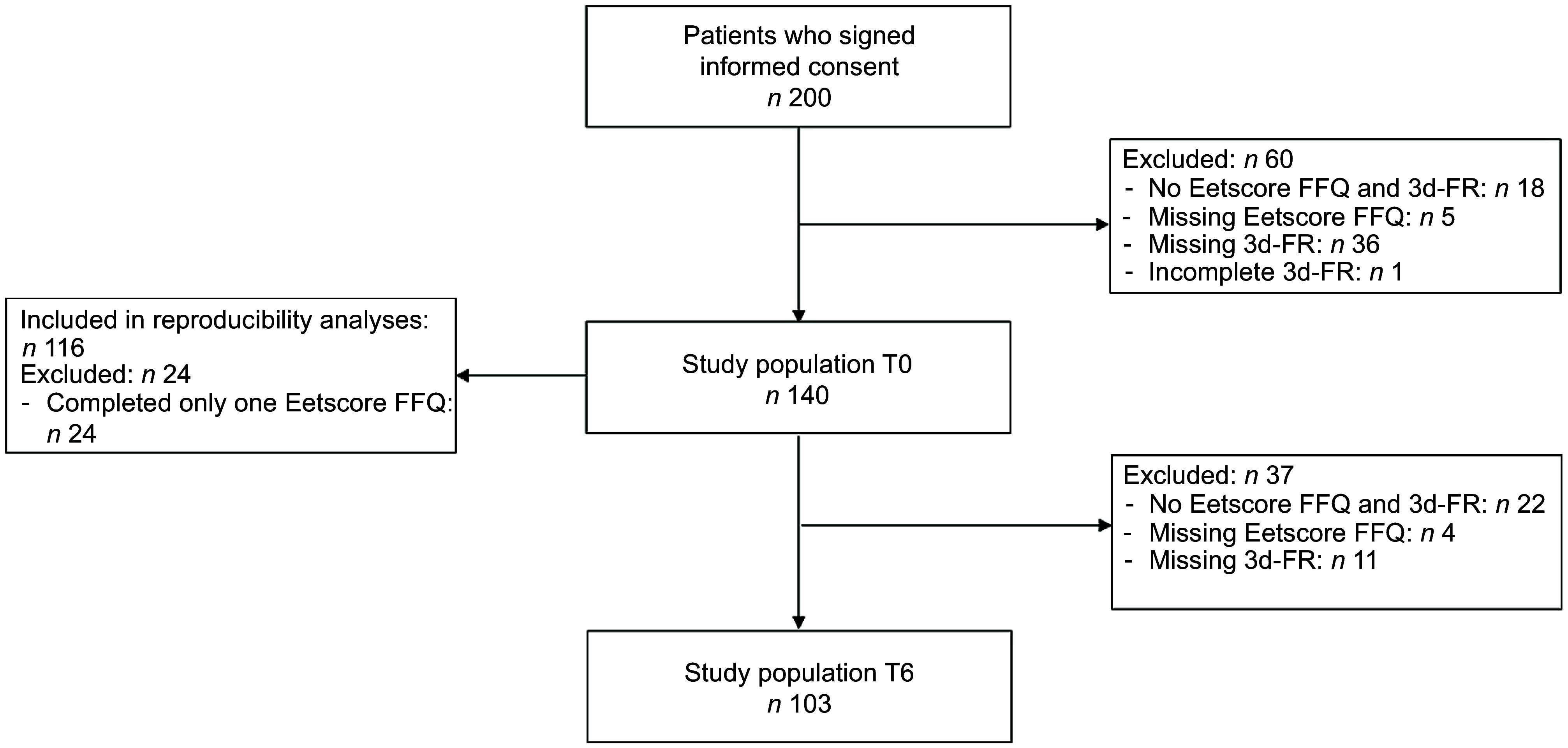
Flow chart of the study population at T0 and T6. 3d-FR, 3-d food records

From the total study sample of 200 participants, we excluded 60 participants with no Eetscore FFQ and 3d-FR (*n* 18), a missing Eetscore FFQ (*n* 5) or a missing/incomplete 3d-FR (*n* 37) at T0. The final study sample for data analysis at T0 consisted of 140 participants, of whom 116 completed both Eetscore FFQ1 and Eetscore FFQ2 (Fig. 1). For the study sample at T6, we additionally excluded 37 participants with no Eetscore FFQ and 3d-FR (*n* 22), a missing Eetscore FFQ (*n* 4) or a missing 3d-FR (*n* 11) at T6, resulting in a final study sample of 103 participants for data analysis at T6 (Fig. 1).

### Data collection

#### Demographic information

Socio-demographic (age, sex and educational level) and health-related information (anthropometrics, type of surgery, comorbidities and smoking status) were obtained from electronic patient records. Educational level was defined as low (primary education and prevocational secondary education), medium (senior general secondary education, pre-university education and secondary vocational education) or high (higher vocational education and university). Anthropometric measurements were performed during standard visits at the hospital. Body weight was measured to the nearest 0·1 kg with a digital weighing scale (Tanita BC-420MA), after removal of heavy clothing and shoes. Height was measured in standing position with a wall-mounted stadiometer (Seca 206). BMI was calculated as weight (kg) divided by squared height (m^2^). TBWL at 6 months was calculated as weight loss divided by body weight before surgery, multiplied by 100 %.

Physical activity at T0 was assessed with the validated Baecke Questionnaire^(17)^ that evaluates a person’s habitual physical activity and separates it into three domains: work index, sports index and leisure index. Each domain could receive a score from 1 to 5 points, resulting in a total score ranging from 3 to 15. A score of 15 indicates being physically active at a high intensity.

#### DHD2015-index

The development of the DHD2015-index has been previously described^(13)^. The DHD2015-index consists of fifteen components representing the Dutch food-based dietary guidelines of 2015^(14)^: vegetables, fruit, wholegrain products, legumes, nuts, dairy, fish, tea, fats and oils, coffee, red meat, processed meat, sweetened beverages, alcohol and Na. Additionally, the component ‘unhealthy food choices’ was added based on the guideline of the Netherlands Nutrition Centre^(18)^. Food items that contributed most to total energy, saturated fat, and mono- and disaccharide intake according to the Dutch National Food Consumption Survey (DNFCS) 2007–2010 were included in this component, such as sweet spreads, pastries, chocolate, savoury snacks, sauces and use of sugar in coffee or tea.

A complete overview of the sixteen components and their cut-off and threshold values is presented in Table 1. For every component, the score ranges from 0 (no adherence) to 10 points (complete adherence), resulting in a total score between 0 and 160 points. A graphic presentation of the scoring of the different types of components can be seen in Supplemental Fig. 1. For adequacy components (vegetables, fruit, legumes, nuts, fish and tea), no intake is awarded with 0 points and intakes between the cut-off and threshold value are scored proportionally. For moderation components (red meat, processed meat, sweetened beverages, Na, alcohol and unhealthy food choices), intakes between the cut-off and threshold value are also scored proportionally, but no intake is awarded with 10 points. Optimum components (dairy) have an optimal range of intake, and ratio components (fat and oils) reflect replacement of less preferred foods (e.g. solid fats) by more preferred foods (e.g. liquid fats and oils). The wholegrain product component is scored based on two sub-components: an adequacy component for wholegrain consumption and a ratio component to reflect replacement of refined grain products by wholegrain products. The coffee component is a qualitative component, based on the type of coffee (filtered *v*. unfiltered). As information on the type of coffee used was not available from the food records, this component could not be included in the validity analyses. For this reason, total score ranged between 0 and 150 for this part of the study.

**Table 1:** Cut-off and threshold values for the calculation of the DHD2015-index components and the component ‘Unhealthy food choices’. Adapted from De Rijk *et al.*^(15)^

	Component	Component type	Dutch dietary guidelines 2015	Minimum score (=0 points)	Maximum score (=10 points)
1	Vegetables	A	Eat at least 200 g of vegetables daily	0 g/d	≥ 200 g/d
2	Fruit	A	Eat at least 200 g of fruit daily	0 g/d	≥ 200 g/d
3	Wholegrain products	A	Eat at least 90 g of wholegrain products daily	0 g/d	≥ 90 g/d
		R	Replace refined cereal products by wholegrain products	No consumption of wholegrain products or ratio of wholegrains to refined grains ≤ 0·7	No consumption of refined products or ratio of wholegrains to refined grains ≥ 11
4	Legumes	A	Eat legumes weekly	0 g/d	≥ 10 g/d
5	Nuts	A	Eat at least 15 g of unsalted nuts daily	0 g/d	≥ 15 g/d
6	Dairy*	O	Eat a few portions of dairy products daily, including milk or yogurt	0 g/d or ≥ 750 g/d	300–450 g/d
7	Fish†	A	Eat one serving of fish weekly, preferably oily fish	0 g/d	≥ 15 g/d
8	Tea	A	Drink three cups of black or green tea daily	0 g/d	≥ 450 ml/d
9	Fats and oils	R	Replace butter, hard margarines and cooking fats by soft margarines, liquid cooking fats and vegetable oils	No consumption of soft margarines, liquid cooking fats and vegetable oils or ratio of liquid cooking fats to solid cooking fats ≤ 0·6	No consumption of butter, hard margarines and cooking fats or ratio of liquid cooking fats to solid cooking fats ≥ 13
10	Coffee	Q	Replace unfiltered coffee by filtered coffee	Any consumption of unfiltered coffee	Consumption of only filtered coffee or no coffee consumption
11	Red meat	M	Limit consumption of red meat	≥ 100 g/d	≤ 45 g/d
12	Processed meat	M	Limit consumption of processed meat	≥ 50 g/d	0 g/d
13	Sweetened beverages and fruit juices	M	Limit consumption of sweetened beverages and fruit juices	≥ 250 g/d	0 g/d
14	Alcohol	M	If alcohol is consumed at all, intake should be limited to one Dutch unit (10 g ethanol) daily	Women: ≥ 20 g ethanol/d Men: ≥ 30 g ethanol/d	Women: ≤ 10 g ethanol/d Men: ≤ 10 g ethanol/d
15	Na	M	Limit consumption of table salt to 6 g daily	≥ 3·8 g Na/d	≤ 1·9 g Na/d
16	Unhealthy food choices	M	Limit consumption of unhealthy choices	≥ 7 week choices/week	≤ 3 week choices/week

DHD2015-index, Dutch Healthy Diet index 2015; A, adequacy component (consume an adequate amount); R, ratio component (replace less healthy products by more healthy alternatives); O, optimum component (optimal consumption range); Q, qualitative component (choose healthier option); M, moderation component (limit consumption).

* Maximum of 40 g/d cheese could be included.

† Maximum of 4 g/d lean fish could be included.

#### The Eetscore FFQ

The development of the Eetscore FFQ has been described in detail elsewhere^(15)^. Briefly, the Eetscore FFQ was developed to assess the DHD2015-index as a measure of adherence to the Dutch food-based dietary guidelines. The Eetscore FFQ assesses dietary intake over the previous month, based on fifty-five food items that account for 85 % of energy intake from the adult population of the DNFCS 2007–2010^(19)^. The six answer categories for questions on frequency of consumption range from ‘never’ to ‘every day’ for regularly consumed foods and from ‘not this month’ to ‘4 times a month’ for episodically consumed foods. Portion sizes are assessed in standard portions and commonly used household measures. Average daily intakes of food items are calculated by multiplying frequency of consumption by portion size in grams. The Eetscore FFQ directly reports index scores of the sixteen components of the DHD2015-index.

#### Three-day food records

A 3-d estimated food record was used as the reference method. This method is considered acceptable for the assessment of usual dietary intake and is commonly used in dietary validation studies^(20)^. We used structured open-ended food records containing predefined food groups (including the option ‘others’) at six food occasions (breakfast, lunch, dinner and three eating occasions between the main meals). All participants received verbal instructions and were provided with a written example. They were asked to record all foods and beverages consumed over the 3 d in as much detail as possible, to describe the amounts consumed in units, household measures or provide weights when known, to report cooking methods and to include the recipes for any mixed dishes. At both time points, recorded days were randomly selected and consisted of 2 weekdays (Monday–Thursday) and 1 weekend day (Friday–Sunday) within a 1-week period. Completed food records were reviewed by the researcher for completeness with regard to portion sizes, cooking methods and description of foods. Telephone interviews with the participants were conducted in case of any uncertainties. Dietary intake data were entered in Compl-eat™, a computer-based nutrition calculation programme that is linked to the Dutch Food Composition Database (NEVO-online, version 2016)^(21)^. All foods and beverages from the food records were categorised into one of the fifteen DHD components (excluding coffee) to calculate the scores of the DHD2015-index. In case of missing recipes for mixed meals such as pasta or rice dishes, standard recipes of the Dutch Food Composition Database (NEVO-online, version 2016) were used^(21)^. Food items that did not fall into one of the DHD components (e.g. potatoes and soups) were not included. Total dietary intake of the fifteen DHD components in grams was averaged over the number of completed days before calculating corresponding index scores.

### Statistical analysis

General characteristics of the study population are reported as medians and interquartile ranges (Q1–Q3) for continuous data and as frequencies and percentages for categorical data. Total DHD2015-index score and individual component scores calculated from the Eetscore FFQ and the 3d-FR are presented as means and standard deviations.

Relative validity of the Eetscore FFQ compared to the 3d-FR was assessed by calculating Kendall’s tau-b (*τ*b) as well as Spearman’s rho (*ρ*) correlation coefficients between the DHD index scores derived from both methods. At T0, we used data of the Eetscore FFQ that was completed in the same month as the 3d-FR. CI for the correlations were obtained using Fisher’s z-transformation. Correlation coefficients less than 0·20 were classified as poor, 0·20–0·49 as acceptable and ≥ 0·50 as good^(22)^. Additionally, total DHD2015-index scores derived from the Eetscore FFQ and the 3d-FR were categorised into tertiles. If ≥ 50 % of the participants were classified into the same tertile and/or ≤ 10 % into the opposite tertile, this was considered a good outcome^(22)^. Weighted kappa coefficients (*k*_*w*_) were calculated to further evaluate the relative level of agreement. *k*_*w*_ coefficients less than 0·20 indicated a poor level of agreement, 0·21–0·40 fair agreement, 0·41–0·60 moderate agreement, 0·61–0·80 good agreement and greater than 0·80 a very good level of agreement^(23)^. Paired *t* tests were used to test the mean differences in the DHD index scores between the two methods. Bland–Altman plots with 95 % limits of agreement were used to visualise the differences in the total DHD2015-index score.

We additionally explored the degree of potential misreporting of dietary intake by comparing reported energy intake calculated from the food records at T0 with energy requirements as identified by the revised Goldberg cut-off method^(24)^. BMR was estimated using the Mifflin-St Jeor Equation^(25)^ as this method provides the best estimation in individuals with (severe) obesity^(26–28)^. We used a physical activity level of 1·55, reflecting a moderate active lifestyle that was in line with the median physical activity score resulting from the Baecke Questionnaire.

Reproducibility of the Eetscore FFQ was examined by calculating single-measures intraclass correlation coefficients (ICC) of absolute agreement between the DHD index scores of both FFQ at T0, using a two-way mixed model. ICC less than 0·50 indicated poor reproducibility, 0·50–0·75 moderate, 0·75–0·90 good and greater than 0·90 excellent reproducibility^(29)^.

All analyses were conducted using SPSS statistics 25.0 (IBM).

## Results

### Participant characteristics

The study population at T0 consisted of 140 participants. The majority was female (79·3 %), never smoked (55·0 %), had a medium educational level (62·8 %) and no comorbidities (51·4 %) (Table 2). Median age was 49·0 (36·5–55·0) years, and median BMI was 41·5 (39·1–45·7) kg/m^2^. Median physical activity score of the Baecke Questionnaire was 8·4 (7·1–9·1).

**Table 2:** Baseline characteristics of the study population at T0 (*n* 140)

	Study population at T0 (*n* 140)	
	Frequencies	Valid percentages
Sex (female)	111	79·3
Age (years)		
Median	49·0	
Q1–Q3	36·5–55·0	
BMI (kg/m^2^)		
Median	41·5	
Q1–Q3	39·1–45·7	
Smoking status		
Never	77	55·0
Former	53	37·9
Current	10	7·1
Educational level*		
Low	24	18·6
Medium	81	62·8
High	24	18·6
Comorbidity		
None	72	51·4
Diabetes mellitus type 2	23	16·4
Dyslipidaemia	25	17·9
Hypertension	43	30·7
OSAS	29	20·7
Physical activity†		
Median	8·4	
Q1–Q3	7·1–9·1	
Adjustable Gastric Band	18	12·9

OSAS, obstructive sleep apnoea syndrome.

* Low education = primary education and prevocational secondary education; medium education = senior general secondary education, pre-university education and secondary vocational education; high education = higher vocational education and university. Missing for *n* 11.

† Based on Baecke Questionnaire; total score ranging from 3 to 15. Missing for *n* 27.

Baseline characteristics of the study population at T6 (*n* 103) were similar to those of the study population at T0 (see online supplementary material, Supplemental Table 1). The majority had undergone a RYGB (80·7 %), and median BMI 6 months after surgery was 30·9 (28·5–34·3) kg/m^2^, resulting in a median TBWL of 25·8 (21·1–29·3) per cent.

### Relative validity of the Eetscore FFQ compared to 3-d food records

**Fig. 2:**
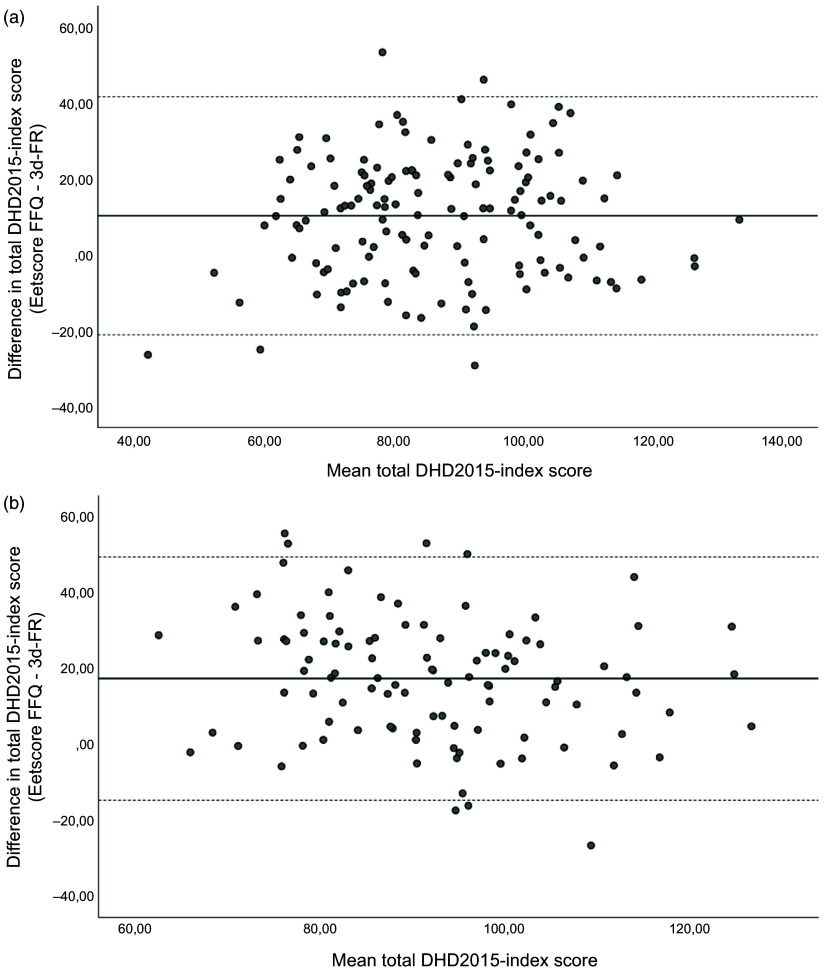
(a) Bland–Altman plot of the total DHD2015-index score derived from the Eetscore FFQ and 3d-FR at T0 (*n* 140). Middle line indicates the mean difference; upper and lower lines indicate limits of agreement based on mean difference ± 1·96 × sd (10·2 ± 31·3). (b) Bland–Altman plot of the total DHD2015-index score derived from the Eetscore FFQ and 3d-FR at T6 (*n* 103). Middle line indicates the mean difference; upper and lower lines indicate limits of agreement based on mean difference ± 1·96 × sd (17·4 ± 32·0). 3d-FR, 3-d food records; DHD2015-index, Dutch Healthy Diet index 2015

Average time difference between completing the Eetscore FFQ and the 3d-FR at T0 was 5·8 ± 7·2 d. Mean total DHD2015-index score derived from the Eetscore FFQ was 10·2 points higher than the score derived from the 3d-FR (91·8 ± 18·6 *v*. 81·5 ± 17·7 points, *P* < 0·001; Table 3a). Visual inspection of the Bland–Altman plot additionally showed relatively wide limits of agreement (–21·1 and 41·5 points, Fig. 2(a)). Index scores for the individual DHD components were significantly different for vegetables, fruit, wholegrain products, legumes, nuts, dairy, fish, tea, processed meat and Na (*P* < 0·05 for all).

**Table 3a:** Mean DHD2015-index scores derived from the 3d-FR and the Eetscore FFQ and corresponding validity statistics in 140 participants before BS (T0)

		3d-FR		Eetscore FFQ		Difference						
		Mean	sd	Mean	sd	Mean	sd	*P*-value	*τ*b	95 % CI	*ρ*	95 % CI
1.	Vegetables	6·7	2·9	5·4	2·8	−1·3	3·3	< 0·001	0·23	0·06, 0·38	0·34	0·18, 0·48
2.	Fruit	7·5	3·4	5·9	3·5	−1·5	3·1	< 0·001	0·41	0·26, 0·54	0·52	0·38, 0·64
3.	Wholegrain products	5·4	2·9	7·1	2·9	+1·7	3·1	< 0·001	0·32	0·16, 0·46	0·42	0·27, 0·55
4.	Legumes	0·8	2·6	5·7	4·5	+4·9	5·1	< 0·001	0·04	−0·13, 0·20	0·05	−0·12, 0·21
5.	Nuts	1·8	3·5	4·0	3·6	+2·2	3·5	< 0·001	0·42	0·27, 0·55	0·50	0·36, 0·62
6.	Dairy	6·9	3·0	6·1	3·3	−0·8	3·8	0·02	0·21	0·04, 0·36	0·28	0·12, 0·43
7.	Fish	2·3	3·8	5·5	3·4	+3·2	4·1	< 0·001	0·31	0·15, 0·46	0·38	0·22, 0·52
8.	Tea	5·0	4·3	4·1	4·3	−0·9	3·9	0·01	0·48	0·33, 0·60	0·57	0·44, 0·68
9.	Fat and oils	6·4	4·5	6·9	4·3	+0·5	5·2	0·26	0·26	0·10, 0·41	0·30	0·14, 0·45
10.	Coffee*	*NA*	*NA*	7·5	2·7	*–*	*–*	*–*	*–*	*–*	*–*	*–*
11.	Red meat	8·9	2·7	8·7	2·7	−0·2	3·7	0·59	0·01	−0·16, 0·18	0·01	−0·16, 0·18
12.	Processed meat	2·2	3·4	3·3	3·1	+1·2	3·5	< 0·001	0·34	0·18, 0·48	0·43	0·28, 0·56
13.	Sweetened beverages	6·6	3·8	7·0	3·8	+0·4	4·0	0·20	0·37	0·21, 0·51	0·47	0·32, 0·60
14.	Alcohol	9·4	2·2	9·3	2·2	−0·1	2·0	0·56	0·54	0·40, 0·65	0·55	0·41, 0·66
15.	Na	7·1	3·2	7·8	2·5	+0·7	3·2	0·01	0·29	0·13, 0·44	0·39	0·23, 0·53
16.	Unhealthy food choices	4·6	4·4	4·8	4·4	+0·2	4·7	0·57	0·34	0·18, 0·48	0·44	0·29, 0·57
	DHD2015-index score†	81·5	17·7	91·8	18·6	+10·2	16·0	< 0·001	0·42	0·27, 0·55	0·60	0·47, 0·70

DHD2015-index, Dutch Healthy Diet index 2015; BS, bariatric surgery; 3d-FR, 3-d food records.

* The component coffee was not assessed in the 3d-FR.

† The total score ranges between 0 and 150 points (excluding coffee component).

Correlation of the total DHD2015-index score was acceptable (*τ*b = 0·42, 95 % CI: 0·27, 0·55), and there was a fair level of agreement between the two methods (*k*_*w*_ = 0·37, 95 % CI: 0·25, 0·49). The Eetscore FFQ correctly classified 50·0 % of the participants into the same tertile as the 3d-FR, and 5·7 % was misclassified into the opposite tertile. For the individual DHD components, a good correlation (≥ 0·50) was observed for alcohol (*τ*b = 0·54, 95 % CI: 0·40, 0·65). Poor correlations (< 0·20) were observed for red meat (*τ*b = 0·01, 95 % CI: –0·16, 0·18) and legumes (*τ*b = 0·04, 95 % CI: –0·13, 0·20). Correlation coefficients of all other components ranged between 0·20 and 0·49.

At T6, average time difference between completing the Eetscore FFQ and the 3d-FR was 8·5 ± 7·4 d. Similar to T0, mean total DHD2015-index score derived from the Eetscore FFQ was higher than from the 3d-FR (mean difference of 17·4 points, *P* < 0·001; Table 3b) with relatively wide limits of agreement (–14·6 and 49·4 points, Fig. 2(b)). Index scores for the individual DHD components were significantly different for vegetables, fruit, wholegrain products, legumes, nuts, fish, fats and oils, processed meat, sweetened beverages and unhealthy food choices (*P* < 0·05 for all).

**Table 3b:** Mean DHD2015-index scores derived from the 3d-FR and the Eetscore FFQ and corresponding validity statistics in 103 participants after BS (T6)

		3d-FR		Eetscore FFQ		Difference						
		Mean	sd	Mean	sd	Mean	sd	*P*-value	*τ*b	95 % CI	*ρ*	95 % CI
1.	Vegetables	4·9	3·0	4·0	2·4	−1·0	3·0	< 0·01	0·27	0·08, 0·44	0·39	0·21, 0·55
2.	Fruit	7·3	3·2	6·4	3·4	−0·9	2·8	< 0·01	0·48	0·31, 0·62	0·60	0·45, 0·72
3.	Wholegrain products	4·4	3·0	6·9	3·0	+2·4	3·5	< 0·001	0·24	0·05, 0·42	0·33	0·14, 0·50
4.	Legumes	1·4	3·5	5·5	4·2	+4·1	5·4	< 0·001	0·07	−0·13, 0·26	0·08	−0·12, 0·27
5.	Nuts	3·0	4·0	4·9	3·5	+2·0	4·1	< 0·001	0·32	0·13, 0·49	0·39	0·21, 0·55
6.	Dairy	6·2	3·7	6·7	3·5	+0·6	4·2	0·17	0·21	0·02, 0·39	0·27	0·08, 0·44
7.	Fish	2·3	3·7	5·9	3·5	+3·7	4·0	< 0·001	0·30	0·11, 0·47	0·36	0·17, 0·52
8.	Tea	4·7	4·4	4·0	4·1	−0·7	3·5	0·06	0·53	0·36, 0·66	0·65	0·51, 0·76
9.	Fat and oils	4·9	4·6	6·2	4·4	+1·3	5·7	0·02	0·17	−0·03, 0·35	0·21	0·02, 0·39
10.	Coffee*	*NA*	*NA*	7·2	2·7	*–*	*–*	*–*	*–*	*–*	*–*	*–*
11.	Red meat	9·2	2·3	9·5	1·8	+0·2	2·6	0·34	0·16	−0·04, 0·34	0·17	−0·03, 0·35
12.	Processed meat	2·9	3·3	5·2	3·0	+2·3	4·0	< 0·001	0·06	−0·14, 0·25	0·09	−0·11, 0·28
13.	Sweetened beverages	6·5	4·0	8·3	2·7	+1·9	4·0	< 0·001	0·25	0·06, 0·43	0·31	0·12, 0·48
14.	Alcohol	9·9	1·0	9·6	1·7	−0·3	1·9	0·18	0·20	0·00, 0·38	0·20	0·00, 0·38
15.	Na	9·1	1·8	9·2	0·5	0·0	1·8	0·90	0·15	−0·05, 0·33	0·17	−0·03, 0·35
16.	Unhealthy food choices	6·6	4·0	8·3	3·0	+1·7	4·5	< 0·001	0·33	0·14, 0·50	0·42	0·24, 0·57
	DHD2015-index score†	83·4	17·2	100·8	14·2	+17·4	16·3	< 0·001	0·31	0·12, 0·48	0·44	0·26, 0·59

DHD2015-index, Dutch Healthy Diet index 2015; BS, bariatric surgery; 3d-FR, 3-d food records.

* The component coffee was not assessed in the 3d-FR.

† The total score ranges between 0 and 150 points (excluding coffee component).

Correlation of the total DHD2015-index score was acceptable (*τ*b = 0·31, 95 % CI: 0·12, 0·48), and there was a fair level of agreement between the two methods (*k*_*w*_ = 0·25, 95 % CI: 0·11, 0·40). The Eetscore FFQ correctly classified 43·7 % of the participants into the same tertile as the 3d-FR, and 9·7 % was misclassified into the opposite tertile. For the individual DHD components, a good correlation (≥ 0·50) was observed for tea (*τ*b = 0·53, 95 % CI: 0·36, 0·66). Poor correlations (< 0·20) were observed for processed meat (*τ*b = 0·06, 95 % CI: –0·14, 0·25), legumes (*τ*b = 0·07, 95 % CI:–0·13, 0·26), Na (*τ*b = 0·15, 95 % CI: –0·05, 0·33), red meat (*τ*b = 0·16, 95 % CI: –0·04, 0·34) and fats and oils (*τ*b = 0·17, 95 % CI: –0·03, 0·35). Correlations coefficients of all other components ranged between 0·20 and 0·49.

### Misreporting

According to the revised Goldberg cut-off method, 57·1 % of the participants was classified as potential under-reporters of energy intake at T0 and 58·3 % of the participants at T6. We did not identify potential over-reporters of energy intake. Excluding potential misreporters did not markedly affect our results regarding the relative validity of the Eetscore FFQ at both time points (see online supplementary material, Supplemental Table 2a and b).

### Reproducibility of the Eetscore FFQ

Average time difference between completing the first and second Eetscore FFQ at T0 was 4·8 ± 2·3 weeks. Mean total DHD2015-index score was 100·4 ± 19·1 points for Eetscore FFQ1 and 103·3 ± 18·3 points for Eetscore FFQ2 (Table 4) with an ICC of 0·78 (95 % CI: 0·69, 0·84). Index scores of the individual DHD components were fairly similar for most components, with ICC ranging from 0·26 to 0·78. Good reproducibility (ICC 0·75–0·90) was observed for fruit (ICC = 0·76, 95 % CI: 0·67, 0·83), fish (ICC = 0·76, 95 % CI: 0·68, 0·83) and coffee (ICC = 0·78, 95 % CI: 0·70, 0·84). Poor reproducibility (ICC < 0·50) was observed for dairy (ICC = 0·26, 95 % CI: 0·08, 0·42), red meat (ICC = 0·29, 95 % CI: 0·11, 0·44), processed meat (ICC = 0·43, 95 % CI: 0·27, 0·57), fats and oils (ICC = 0·46, 95 % CI: 0·30, 0·59) and sweetened beverages (ICC = 0·46, 95 % CI: 0·30, 0·59). ICC of all other components ranged between 0·50 and 0·75 (Table 4).

**Table 4:** Mean DHD2015-index scores derived from the first and second Eetscore FFQ and corresponding intraclass correlation coefficients (ICC) in 116 participants before BS (T0)

		Eetscore FFQ1		Eetscore FFQ2			
		Mean	sd	Mean	sd	ICC	95 % CI
1.	Vegetables	5·7	2·8	5·1	2·8	0·54	0·40, 0·66
2.	Fruit	6·0	3·5	6·5	3·3	0·76	0·67, 0·83
3.	Wholegrain products	7·2	2·8	7·6	2·7	0·70	0·59, 0·78
4.	Legumes	5·7	4·6	6·2	4·4	0·62	0·49, 0·72
5.	Nuts	4·3	3·7	4·1	3·5	0·71	0·61, 0·79
6.	Dairy	6·2	3·3	6·4	3·4	0·26	0·08, 0·42
7.	Fish	5·3	3·4	5·4	3·5	0·76	0·68, 0·83
8.	Tea	4·0	4·2	3·4	3·9	0·68	0·56, 0·76
9.	Fat and oils	7·2	4·2	6·5	4·4	0·46	0·30, 0·59
10.	Coffee	7·4	2·8	7·5	2·7	0·78	0·70, 0·84
11.	Red meat	8·8	2·5	9·0	2·4	0·29	0·11, 0·44
12.	Processed meat	3·3	3·1	3·7	3·3	0·43	0·27, 0·57
13.	Sweetened beverages	7·2	3·7	7·9	3·1	0·46	0·30, 0·59
14.	Alcohol	9·3	2·6	9·4	2·3	0·74	0·65, 0·82
15.	Na	8·0	2·4	8·5	1·9	0·55	0·41, 0·67
16.	Unhealthy food choices	4·9	4·3	6·2	4·2	0·60	0·45, 0·72
	DHD2015-index score*	100·4	19·1	103·3	18·3	0·78	0·69, 0·84

DHD2015-index, Dutch Healthy Diet index 2015; BS, bariatric surgery.

* The total score ranges between 0 and 160 points.

## Discussion

In this study, we determined the relative validity and reproducibility of the Eetscore FFQ as a screener for diet quality in patients with (severe) obesity before and after BS by comparing index scores of the DHD2015-index derived from the Eetscore FFQ to the scores derived from 3d-FR (reference method). We demonstrated an overall reasonable relative agreement between the two methods, although the Eetscore FFQ showed higher index scores in comparison with the 3-FR and absolute agreement between the two methods was poor. Correlation coefficients for the DHD component scores varied widely with best coefficients observed for fruit and tea, and worst for legumes and red meat. Reproducibility of the Eetscore FFQ was considered good.

We observed lower correlations for the total DHD2015-index score based on fifteen components (excluding coffee) between the Eetscore FFQ and 3d-FR than reported in the study of de Rijk et al., who compared the Eetscore FFQ to a full-length FFQ^(15)^. They reported a Kendall’s tau-b coefficient of 0·51 (95 % CI: 0·47, 0·55) for the total DHD2015-index score based on thirteen DHD components (excluding fish, fats and oils, and coffee). This could be explained by a difference in the number of DHD components included in the total score as well as a difference in reference method. The Eetscore FFQ is also an FFQ; therefore, more correlated errors might be expected with a full-length FFQ, resulting in higher correlations. Yet, a full-length FFQ might capture habitual dietary intake more accurately than three food records. Although all days of the week were equally represented across all records, foods that are not consumed on a daily basis, for example fish or legumes, could have been underestimated when recording only 3 d. This is also reflected in relative large absolute differences for these components. It has been suggested that when dietary methods assessing habitual dietary intake, such as the Eetscore FFQ, are validated against food records, a certain degree of disagreement can be expected due to the greater within-subject variations that occur over the shorter reference period of a food record^(20)^.

In a study of Papadaki et al., Pearson’s correlation coefficient of 0·52 was observed comparing the English version of the ‘Mediterranean Diet Adherence Screener’ to 3d-FR in patients with high cardiovascular risk in the UK^(30)^. Schröder et al. found Pearson’s correlation coefficient of 0·61 when they compared the ‘Diet Quality Index’ derived from the ‘Short Diet Quality Screener’ to ten 24-h dietary recalls in a Spanish population^(31)^. In the same study, they also observed a correlation of 0·40 for the ‘Modified Mediterranean Diet Score’ derived from the ‘Brief Mediterranean Diet Screener’ compared with the score derived from ten 24-h dietary recalls^(31)^. These values are comparable to Spearman’s Rho correlations observed in the current study (*ρ* = 0·60, 95 % CI: 0·47, 0·70 at T0 and *ρ* = 0·44, 95 % CI: 0·26, 0·59 at T6).

In contrast to the findings on relative agreement, absolute agreement between the Eetscore FFQ and the 3d-FR was poor. According to the Bland–Altman plots, the Eetscore FFQ systematically overestimated the total DHD2015-index score compared to the 3d-FR at both time points with relatively wide limits of agreement. However, no significant proportional bias was observed. This is in line with other studies that also found higher mean index scores derived from a diet screener in comparison with food records^(15,30–32)^.

As most FFQ, the Eetscore FFQ can be considered more appropriate for ranking patients according to their diet quality or monitoring relative differences over time, rather than assessing absolute individual scores. It is however important to note that a food record is also no golden reference method and has its own limitations with regard to assessing dietary intake. Furthermore, we evaluated the intake of food groups instead of nutrients which is more difficult because of the high day-to-day variation. This may have impacted our findings with respect to the poor absolute agreement between the two methods.

With regard to the individual DHD components, correlations varied widely with highest values found for fruit and tea, and lowest values for legumes and red meat. For legumes, we observed many participants with an extreme difference of 10 points between the index score derived from the Eetscore FFQ compared to the food record-derived score, meaning that these participants had a score of 10 for legumes according to the Eetscore FFQ, whereas their score was 0 based on the food records. This resulted in large mean differences for this component (5·7 *v*. 0·8 points at T0 and 5·5 *v*. 1·4 points at T6, *P* < 0·001). This could be due to the fact that food records might not accurately capture habitual dietary intake, especially for foods that are not consumed on a daily basis such as legumes, as mentioned earlier. This is in concordance with an Australian study (age ≥ 70) validating a six-item dietary screener against three 24-h dietary recalls that also observed a poor agreement for legume intake (*k*_*w*_ = 0·12)^(33)^.

For red meat, we observed poor correlations of < 0·20 at both time points, whereas mean index scores for this component were fairly similar between the two methods (8·7 *v*. 8·9 points at T0 and 9·5 *v*. 9·2 points at T6, *P* > 0·05). This might be explained by a low variation in the index scores for red meat. Over half of the participants scored 10 points based on the Eetscore FFQ as well as the 3d-FR. As a result, the few observations with (relatively) large differences in index score could have biased the correlation towards zero.

We also aimed to define participants who substantially under- or overreported their dietary intake by using the revised Goldberg cut-off method in which energy intake is compared with (estimated) energy expenditure. However, adequately estimating energy expenditure in subjects with (severe) obesity is challenging. In a study of Cancello et al.^(26)^, predictive equations for resting energy expenditure were compared to indirect calorimetry in 4247 subjects with obesity (69 % women, mean age 48 ± 19 years, mean BMI 44 ± 7 kg/m^2^). The authors found that the Mifflin-St Jeor equation had the highest performance for both accuracy and bias but emphasise that the accuracy is still far from ideal^(26)^. Furthermore, the revised Goldberg cut-off method cannot be applied after BS as the condition of weight stability is violated, resulting in an invalid ratio between reported energy intake and energy requirement. We therefore assumed that participants who were identified as potential misreporters of dietary intake at T0 also misreported their intake at T6.

At both time points, the rate of potential misreporters was relatively high with 57·1 % of the study population potentially underreporting their dietary intake at T0 and 58·3 % at T6. According to a review of Poslusna et al., the percentage of under-reporters in studies using estimated food records ranged from 12 to 44 %^(34)^, which is lower than the observed percentages in the present study. This is in line with previous research showing that a higher BMI is associated with underreporting of dietary intake^(35)^.

Overall, excluding potential misreporters did not markedly affect our results, although caution is needed in the interpretation because of the aforementioned limitations in the use of the Goldberg cut-off method within this population.

Reproducibility of the Eetscore FFQ before surgery was considered good. The observed ICC of 0·78 was slightly lower than reported in previous research by de Rijk et al., who found an ICC of 0·91 for the total DHD2015-index score^(15)^. This could be due to a difference in study population as well as the multidisciplinary lifestyle programme that all participants started before undergoing BS. During this programme, patients received general information on healthy eating behaviour and dietary counselling. For most participants, the first Eetscore FFQ was administered before entering the multidisciplinary programme while they completed the second Eetscore FFQ during the programme. It is therefore plausible that participants already implemented beneficial changes with respect to their diet. This might explain the slightly higher DHD2015-index score resulting from the second Eetscore FFQ. Future studies are needed to confirm our findings while limiting the influence of such external factors. For the individual DHD components, most correlation coefficients ranged between 0·5 and 0·7 which are common in reproducibility studies of FFQ^(20)^.

Dietary assessment is an important component in the BS programme. Currently, dietary intake of patients undergoing BS is often assessed by a dietitian with the use of food records. This assessment method is very time-consuming, might be prone to reactivity and recall bias and only reflects the intake of the past days. The Eetscore FFQ is a short, web-based tool that can be used to assess general aspects of a healthy nutrient-dense diet such as the consumption of fruits and vegetables, wholegrains and dairy. However, the Eetscore FFQ does not include additional information about patients’ eating behaviour including the distribution of food intake (e.g. few large meals or frequent smaller feedings) and the separation of food and beverages. Also, other factors affecting dietary intake may be missed by the Eetscore FFQ, such as food preparation methods and non-included food items (e.g. plant-based dairy, meat substitutes and fast food). The Eetscore FFQ can therefore be used as an additional dietary assessment tool in the BS programme rather than as a replacement for the current methodology.

Considering the need for dietary assessment methods that reduce the burden for patients, practitioners and researchers, the Eetscore FFQ can be used for ranking patients according to diet quality and for monitoring relative changes in intake over time in order to indicate an improvement or a deterioration in diet quality. This can be relevant before undergoing surgery, during annual follow-up in the late post-operative phase or in case of weight regain. Dietary assessment methods assessing actual intake may be preferred in the early post-operative phase when patients are still adapting to the new eating habits and in case of food-related complaints such as dumping syndrome or hypoglycaemia.

The main strength of this study is the validation of an existing dietary assessment tool in patients with (severe) obesity before and after BS as there is a clear lack of validated, easy-to-use tools within this patient population. Another strength is the use of multiple statistical tests to provide a comprehensive insight into various facets of validity. As Kendall’s tau-b correlation coefficients tend to be smaller, we also reported Spearman’s Rho correlations to allow for comparison with other research. Furthermore, by choosing 3d-FR as reference method, we minimised the risk of correlated measurement errors between the two methods^(20)^.

We aimed to determine relative validity of the Eetscore FFQ both before and after BS, but thirty-seven participants dropped out between T0 and T6, resulting in two different study populations. We are aware that the study population at T0 and T6 is therefore not mutually exclusive and direct comparisons between the populations cannot be made. Nonetheless, both populations and the dropouts were similar with respect to sex, age, BMI, smoking status, education, physical activity, prevalence of comorbidities and type of surgery. Moreover, both the study population at T0 and T6 were found representative of the general Dutch bariatric patient population^(36)^, indicating a minor risk of selection bias.

Another limitation is the lack of a golden standard reference method for dietary intake. To reduce participant burden, we chose for 3d-FR using household measures, which are prone to report bias and are not ideal for foods that are not consumed daily. For future research, we suggest to evaluate the Eetscore FFQ against dietary biomarkers that are suitable for patients after BS to provide an objective measure of dietary intake.

## Conclusion

The Eetscore FFQ is a short screener of diet quality that assesses adherence to the Dutch dietary guidelines. Based on our findings, the Eetscore FFQ was considered an acceptable screener for ranking individuals according to their diet quality and showed good reproducibility to monitor relative changes in diet quality over time. However, the tool showed poor absolute agreement and is not suitable for assessing diet quality on the individual level. Future research is needed to improve the use of the Eetscore FFQ for this purpose.

## Supplementary material

For supplementary material accompanying this paper visit https://doi.org/10.1017/S1368980022001501. click here to view supplementary material
