# Supplementary material for: Relative validity of a short screener to assess diet quality in patients with severe obesity before and after bariatric surgery
Source: Public Health Nutr. 2022 Jul 4;25(10):2731–41. doi: 10.1017/S1368980022001501 (PMC9991825; doi:10.1017/S1368980022001501)
Supplement: Supplementary file 1 [file S1368980022001501sup001.docx]

**Supplementary Table 1.**  Baseline characteristics of the study population at T6

|  | **Study population at T6**  (n=103) | |
| --- | --- | --- |
| **Sex** (female) | 81 | (78.6) |
| **Age** (years ) | 50.0 | [41.0-56.0] |
| **BMI** (kg/m²) | 41.6 | [39.3-45.0] |
| **Smoking status** |  |  |
| Never | 61 | (59.2) |
| Former | 37 | (35.9) |
| Current | 5 | (4.9) |
| **Educational level*** |  |  |
| Low | 16 | (17.0) |
| Medium | 61 | (64.9) |
| High | 17 | (18.1) |
| **Comorbidity** |  |  |
| None | 52 | (50.5) |
| Diabetes Mellitus type 2 | 18 | (17.5) |
| Dyslipidaemia | 21 | (20.4) |
| Hypertension | 33 | (32.0) |
| OSAS | 18 | (17.5) |
| **Physical activity** † | 8.4 | [7.1-8.9] |
| **Adjustable Gastric Band** | 15 | (14.6) |

Data are presented as medians [Q1-Q3] and frequencies (valid percentages).

*BMI*, Body Mass Index. *OSAS*, Obstructive Sleep Apnoea Syndrome.

* Low education = primary education and prevocational secondary education; medium education = senior general secondary education, pre-university education and secondary vocational education; high education = higher vocational education and university. Missing for n=9.

† Based on Baecke questionnaire; total score ranging from 3-15. Missing for n=21.

**Supplementary Table 2a**. Mean DHD2015-index scores derived from the 3d-FR and the Eetscore FFQ and corresponding validity statistics in 60 participants before BS (T0). Potential under-reporters of energy intake are excluded (n=80).

|  |  | 3d-FR | | Eetscore FFQ | | Difference | | | | |  |  |  |  |
| --- | --- | --- | --- | --- | --- | --- | --- | --- | --- | --- | --- | --- | --- | --- |
|  |  | Mean | SD | Mean | SD |  | Mean | SD | P-value |  | τb | 95% CI | ρ | 95% CI |
| 1. | Vegetables | 7.0 | 2.9 | 5.3 | 2.6 |  | -1.7 | 3.4 | <0.001 |  | 0.14 | -0.12, 0.38 | 0.20 | -0.06, 0.43 |
| 2. | Fruit | 7.6 | 3.4 | 5.8 | 3.6 |  | -1.9 | 3.2 | <0.001 |  | 0.41 | 0.16, 0.61 | 0.51 | 0.28, 0.69 |
| 3. | Wholegrain products | 5.4 | 2.9 | 6.8 | 2.9 |  | +1.4 | 3.1 | <0.001 |  | 0.35 | 0.10, 0.56 | 0.45 | 0.21, 0.64 |
| 4. | Legumes | 0.7 | 2.5 | 5.5 | 4.5 |  | +4.9 | 5.3 | <0.001 |  | -0.07 | -0.32, 0.19 | -0.08 | -0.33, 0.18 |
| 5. | Nuts | 2.1 | 3.7 | 4.2 | 3.6 |  | +2.0 | 3.8 | <0.001 |  | 0.39 | 0.14, 0.59 | 0.46 | 0.22, 0.65 |
| 6. | Dairy | 6.5 | 3.3 | 6.2 | 3.3 |  | -0.4 | 3.4 | 0.37 |  | 0.32 | 0.07, 0.54 | 0.43 | 0.19, 0.62 |
| 7. | Fish | 1.9 | 3.6 | 5.3 | 3.2 |  | +3.5 | 4.0 | <0.001 |  | 0.29 | 0.03, 0.51 | 0.36 | 0.11, 0.57 |
| 8. | Tea | 5.1 | 4.3 | 4.0 | 4.3 |  | -1.0 | 3.9 | 0.04 |  | 0.52 | 0.29, 0.69 | 0.61 | 0.40, 0.76 |
| 9. | Fat and oils | 6.0 | 4.5 | 6.5 | 4.4 |  | +0.5 | 5.1 | 0.43 |  | 0.28 | 0.02, 0.50 | 0.34 | 0.09, 0.55 |
| 10. | Coffee* | *NA* | *NA* | 7.4 | 2.8 |  | *-* | *-* | *-* |  | *-* | - | - | - |
| 11. | Red meat | 8.6 | 3.0 | 8.4 | 3.3 |  | -0.2 | 4.5 | 0.75 |  | -0.04 | -0.29, 0.22 | -0.05 | -0.30, 0.21 |
| 12. | Processed meat | 1.4 | 2.9 | 3.0 | 2.9 |  | +1.5 | 3.2 | <0.001 |  | 0.32 | 0.07, 0.54 | 0.39 | 0.14, 0.59 |
| 13. | Sweetened beverages | 5.9 | 4.2 | 7.1 | 3.8 |  | +1.2 | 3.9 | 0.02 |  | 0.41 | 0.16, 0.61 | 0.50 | 0.27, 0.68 |
| 14. | Alcohol | 9.1 | 2.7 | 9.1 | 2.4 |  | 0.0 | 2.2 | 0.90 |  | 0.58 | 0.36, 0.74 | 0.60 | 0.39, 0.75 |
| 15. | Sodium | 5.9 | 3.3 | 7.2 | 3.0 |  | +1.4 | 3.8 | 0.01 |  | 0.26 | 0.00, 0.49 | 0.33 | 0.08, 0.54 |
| 16. | Unhealthy food choices | 3.3 | 4.2 | 3.4 | 4.3 |  | +0.1 | 4.7 | 0.91 |  | 0.36 | 0.11, 0.57 | 0.44 | 0.20, 0.63 |
|  | DHD2015-index score† | 76.5 | 16.9 | 87.8 | 17.0 |  | +11.3 | 15.8 | <0.001 |  | 0.39 | 0.14, 0.59 | 0.57 | 0.35, 0.73 |

*3d-FR*, three day food records.

* The component coffee was not assessed in the 3d-FR.

† The total score ranges between 0 and 150 points (excluding coffee component).

**Supplementary Table 2b**. Mean DHD2015-index scores derived from the 3d-FR and the Eetscore FFQ and corresponding validity statistics in 43 participants after surgery (T6). Potential under-reporters of energy intake are excluded (n=60).

|  |  | 3d-FR | | Eetscore FFQ | | Difference | | | | |  |  |  |  |
| --- | --- | --- | --- | --- | --- | --- | --- | --- | --- | --- | --- | --- | --- | --- |
|  |  | Mean | SD | Mean | SD |  | Mean | SD | P-value |  | τb | 95% CI | ρ | 95% CI |
| 1. | Vegetables | 5.0 | 3.1 | 4.0 | 2.4 |  | -1.0 | 3.1 | 0.05 |  | 0.27 | -0.04, 0.53 | 0.37 | 0.07, 0.61 |
| 2. | Fruit | 7.7 | 2.8 | 6.8 | 3.3 |  | -0.9 | 2.3 | 0.01 |  | 0.52 | 0.24, 0.72 | 0.64 | 0.40, 0.80 |
| 3. | Wholegrain products | 4.6 | 2.9 | 7.3 | 3.3 |  | +2.7 | 3.4 | <0.001 |  | 0.28 | -0.03, 0.54 | 0.39 | 0.09, 0.63 |
| 4. | Legumes | 1.2 | 3.2 | 5.2 | 4.1 |  | +4.1 | 5.2 | <0.001 |  | 0.02 | -0.28, 0.32 | 0.02 | -0.28, 0.32 |
| 5. | Nuts | 2.7 | 3.7 | 4.6 | 3.8 |  | +1.8 | 4.4 | 0.01 |  | 0.23 | -0.08, 0.50 | 0.28 | -0.03, 0.54 |
| 6. | Dairy | 6.6 | 3.5 | 6.9 | 3.2 |  | +0.4 | 4.5 | 0.61 |  | 0.18 | -0.13, 0.46 | 0.24 | -0.07, 0.51 |
| 7. | Fish | 2.7 | 3.9 | 5.8 | 3.3 |  | +3.2 | 3.8 | <0.001 |  | 0.37 | 0.07, 0.61 | 0.43 | 0.14, 0.65 |
| 8. | Tea | 4.8 | 4.6 | 4.4 | 4.3 |  | -0.4 | 2.8 | 0.32 |  | 0.65 | 0.41, 0.81 | 0.76 | 0.57, 0.87 |
| 9. | Fat and oils | 4.8 | 4.5 | 6.0 | 4.4 |  | +1.2 | 6.0 | 0.18 |  | 0.13 | -0.18, 0.42 | 0.30 | -0.01, 0.56 |
| 10. | Coffee* | *NA* | *NA* | 7.3 | 2.5 |  | *-* | *-* | *-* |  | - | - | - | - |
| 11. | Red meat | 9.2 | 2.5 | 9.7 | 1.3 |  | +0.5 | 2.3 | 0.17 |  | 0.17 | -0.14, 0.45 | 0.26 | -0.05, 0.52 |
| 12. | Processed meat | 2.4 | 3.3 | 4.8 | 2.6 |  | +2.5 | 3.2 | <0.001 |  | 0.22 | -0.09, 0.49 | 0.31 | 0.00, 0.56 |
| 13. | Sweetened beverages | 6.5 | 4.0 | 8.3 | 2.6 |  | +1.7 | 4.1 | 0.01 |  | 0.26 | -0.05, 0.52 | 0.29 | -0.02, 0.55 |
| 14. | Alcohol | 9.8 | 1.5 | 9.7 | 1.6 |  | -0.1 | 2.2 | 0.75 |  | -0.06 | -0.35, 0.25 | -0.06 | -0.35, 0.25 |
| 15. | Sodium | 8.6 | 2.3 | 9.2 | 0.5 |  | +0.6 | 2.2 | 0.10 |  | 0.09 | -0.22, 0.38 | 0.11 | -0.20, 0.40 |
| 16. | Unhealthy food choices | 4.8 | 4.2 | 8.1 | 2.9 |  | +3.3 | 4.9 | <0.001 |  | 0.22 | -0.09, 0.49 | 0.29 | -0.02, 0.55 |
|  | DHD2015-index score† | 81.2 | 17.6 | 100.8 | 13.6 |  | +19.6 | 16.5 | <0.001 |  | 0.28 | -0.03, 0.54 | 0.40 | 0.10, 0.63 |

*3d-FR*, three day food records.

* The component coffee was not assessed in the 3d-FR.

† The total score ranges between 0 and 150 points (excluding coffee component).

**Supplementary Figure 1.** Graphic presentation of scoring of the different types of components: adequacy component (A), moderation component (B), optimum component (C) and ratio component. *Adapted from Looman et al* ^(13)^

*
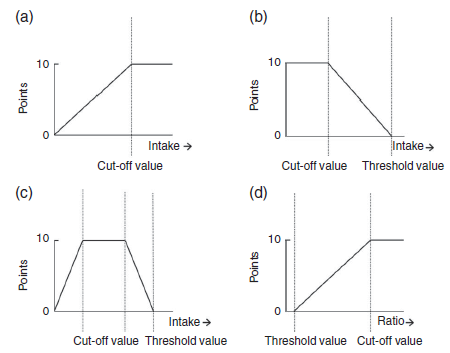
*
